# Experimental osteoarthritis model by means of medial meniscectomy in rats and effects of diacerein administration and hyaluronic acid injection: Modelo experimental de osteoartrite através da meniscectomia medial em ratos e efeitos da administração de diacereína e da injeção de ácido hialurônico

**DOI:** 10.1590/1516-3180.2013.6730001

**Published:** 2014-11-28

**Authors:** Marcia Uchôa Rezende, Arnaldo José Hernandez, Claudia Regina Gomes Cardim Mendes Oliveira, Raul Bolliger

**Affiliations:** I MD, PhD. Collaborating Professor, Department of Orthopedics and Traumatology, Faculdade de Medicina, Universidade de São Paulo (FMUSP), São Paulo, Brazil.; II MD, PhD. Associate Professor, Department of Orthopedics and Traumatology, Faculdade de Medicina, Universidade de São Paulo (FMUSP), São Paulo, Brazil.

**Keywords:** Osteoarthritis, Knee, Anthraquinones, Hyaluronic acid, Models, animal

## Abstract

**CONTEXT AND OBJECTIVE:**

The development of a slow and progressive mechanical model for osteoarthritis is important for correlation with clinical practice, and for evaluating the effects of disease-modifying medications. A mechanical osteoarthritis model was developed to evaluate the effects of intra-articular hyaluronic acid (HA) injection and oral diacerein administration.

**DESIGN AND SETTING:**

Experimental study at the Department of Orthopedics and Traumatology, Universidade de São Paulo.

**METHOD:**

Total medial meniscectomy was performed on seven groups of ten Wistar rats each, comprising four control groups (C) and three study groups (S). C.I: operated, non-medicated; C.II: operated, injections of HA vehicle; C.III: non-operated, non-medicated; C.IV: operated, non-medicated, sacrificed three months post-meniscectomy; S.I: operated, receiving intra-articular HA injections; S.II: operated, oral diacerein from the third to the seventh postoperative month; S.III: operated, received both medications. All the animals (except C.IV) were sacrificed seven months post-meniscectomy. All femurs and tibias were assessed histologically.

**RESULTS:**

The most severe degenerative histological changes were in the tibias of the operated knees. On the contralateral side, all groups had mild changes on the tibial surface. The femoral surface had slight changes. C.I showed severe changes. S.II results matched those of C.IV. HA protected the tibial surface. S.II and S.III had similar results.

**CONCLUSIONS:**

1) The experimental model produced mild arthritis after three months and severe arthritis after seven months; 2) diacerein reduced the degenerative changes in both knees; 3) HA protected the joint cartilage; 4) Combining the two drugs did not improve the results.

## RESUMO

**CONTEXTO E OBJETIVO:**

Desenvolver um modelo osteoartrítico mecânico lento e progressivo é importante para correlação com a prática clínica e para avaliar os efeitos de medicamentos modificadores da doença. Um modelo mecânico de osteartrite foi desenvolvido para avaliar os efeitos de injeção intra-articular de hialuronato de sódio (AH) e de administração de diacereína oral.

**DESENHO E LOCAL:**

Estudo experimental no Departamento de Ortopedia e Traumatologia, Universidade de São Paulo.

**MÉTODO:**

Meniscectomia medial total foi feita em sete grupos de dez ratos Wistar, sendo quatro grupos controle (C) e três grupos estudo (E). C.I: operado, não medicado; C.II: operado, recebendo injeções do veículo do AH; C.III: não operado, não medicado; C.IV: operado, não medicado, sacrificado três meses pós-meniscectomia; EI: operado, recebendo injeções de AH intra-articular; E.II: operado, recebendo diacereína oral do terceiro ao sétimo mês pós-operatório; E.III: operado, recebeu ambas medicações. Todos os animais (exceto C.IV) foram sacrificados sete meses pós-meniscectomia. Todos os fêmures e tíbias foram analisados histologicamente.

**RESULTADOS:**

As alterações histológicas degenerativas mais graves encontram-se nas tíbias dos joelhos operados. No lado contra-lateral, todos os grupos apresentaram alterações leves na superfície tibial. A superfície femoral teve ligeiras alterações. C.I apresentou alterações graves. S.II equiparou seus resultados aos do grupo C.IV. AH protegeu a superfície tibial. S.II e S.III apresentaram resultados semelhantes.

**CONCLUSÕES:**

1) O modelo experimental gerou artrite leve aos três e grave aos sete meses; 2) Diacereína reduziu as alterações degenerativas de ambos os joelhos; 3) AH protege a cartilagem articular; 4) A associação dos dois fármacos não melhora os resultados.

## INTRODUCTION

Osteoarthritis (OA) is classified as primary, idiopathic or secondary.^1^ Primary OA is either localized or generalized, based on whether less than three or three or more joints are affected, respectively.^1^ Idiopathic knee OA can be also divided according to the affected site into medial, lateral and/or patellofemoral compartment OA.^1^ Secondary OA is clearly a consequence of an earlier injury to the joint.^2^ The fact that the pathological changes observed are similar in the two basic types of OA suggests that the final biochemical pathways that result in cartilage degeneration may also be similar. If so, pharmacological intervention directed toward basic biochemical malfunctions may be possible in all forms of OA.^2^

Analgesics and non-steroidal anti-inflammatory drugs (NSAIDs) are used in accordance with the guidelines for treatment of osteoarthritis;^3^ however, these medications do not prevent degenerative changes. NSAIDs have been correlated with serious adverse effects, and particularly renal and gastrointestinal effects. Selective cyclooxygenase-2 (COX-2) inhibitors reduce the incidence of upper gastrointestinal tract ulcerations; however, their side effects include fluid retention, hypertension, congestive heart failure, renal insufficiency and a risk of cardiovascular thrombosis.^4^

On the other hand, it is claimed that drugs known as slow-acting disease-modifying drugs relieve pain and slow down the progression of osteoarthritis.^4^ Among these are intermittent intra-articular injection of hyaluronic acid and continuous oral intake of diacerein. Evidence of reduced degenerative changes has been shown with both of these medications.^4^^,^^5^^,^^6^

Although experimental models for osteoarthritis may have their limitations and criticisms, OA-like pathological changes have been described in the knee joints of several species of animals.^2^ These models have mainly consisted of mice, rabbits, dogs and sheep.^2^^,^^7^^,^^8^^,^^9^^,^^10^^,^^11^ Sectioning of the anterior cruciate ligament^9^^,^^10^ and partial or total meniscectomy in dogs,^12^ rabbits^2^^,^^13^^,^^14^^,^^15^ and sheep^5^^,^^11^ have frequently been used as models for osteoarthritis. Less frequently used, but more aggressive models for osteoarthritis have been produced through combining ligament injury with meniscectomy in rabbits.^2^^,^^14^^,^^15^

In view of the possibility that drug therapy may delay the development of this condition, we proposed an experimental model for osteoarthritis consisting of medial meniscectomy in rats, in order to register the degenerative changes and investigate the effects of so-called slow-acting disease-modifying drugs. Specifically in this study, we evaluated the two drugs mentioned above (oral diacerein and intra-articular hyaluronic acid) for reducing the development of osteoarthritis. The drugs were administered in the early stages of the disease either individually or in combination.

## OBJECTIVE

The primary objective was to develop an experimental model for osteoarthritis in rats, by means of medial meniscectomy.

The secondary objective was to investigate the effects of two drugs, diacerein (orally) and hyaluronic acid (intra-articular), separately and in association, when administered in the early stages of the disease caused by the experimental model for OA.

## METHODS

This study was approved by the local Ethics Committee (CAPPesq), under the number 690/98. The study was conducted at LIM-41, IOT-HC-USP.

Seventy adult male Wistar rats (age range from 8 to 16 weeks and weight range from 180 to 360 grams) were randomly assigned to four control groups (C) and three study groups (S):

- C.I - Ten rats were subjected to total medial meniscectomy of the right knee and were sacrificed 28 weeks (seven months) post-surgery.
- C.II - Ten rats were subjected to total medial meniscectomy of the right knee and were injected with the hyaluronic acid vehicle; they were sacrificed 28 weeks (seven months) post-surgery.
- C.III - Ten rats were not subjected to any procedure, and were sacrificed 28 weeks (seven months) after inclusion.
- C.IV - Ten rats were subjected to total medial meniscectomy of the right knee and were sacrificed twelve weeks (three months) post-surgery.
- S.I - Ten rats were subjected to total medial meniscectomy of the right knee and were injected with hyaluronic acid; they were sacrificed 28 weeks (seven months) post-surgery.
- S.II - Ten rats were subjected to medial meniscectomy of the right knee and received oral diacerein; they were sacrificed 28 weeks (seven months) post-surgery.
- S.III - Ten rats were subjected to medial meniscectomy of the right knee and received both oral diacerein and injected hyaluronic acid; they were sacrificed 28 weeks (seven months) post-surgery.

All the rats with the exception of those in group C.III, which were not subjected to any operation, received intra-peritoneal anesthesia and the following procedures were performed.

**Figure 1.**
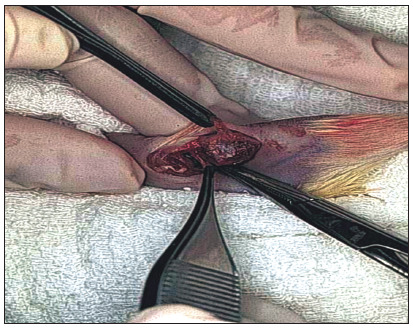
Medial meniscectomy by means of a medial access in a Wistar rat. Tibial collateral ligament is sectioned 3 mm from the joint line and retracted to expose the medial meniscus. After meniscectomy, the ligament is sutured with nylon 5-0.

A 3-cm longitudinal incision was made on the medial aspect of the right knee. By retracting the quadriceps muscle anteriorly, the tibial collateral ligament was exposed and sectioned 3 mm from the joint line, and was then retracted. The medial meniscus was extracted (Figure 1). The tibial collateral ligament was sutured with nylon 5-0. The quadriceps and skin were sutured with nylon 4-0. The wound was cleansed using saline and chlorhexidine gluconate solution.

After the animals had recovered from the anesthesia, they received 2.5 mg of oral dipyrone. After surgery, the animals were kept in standard cages (three animals/cage).

Four times a week, the animals were stimulated to walk up and down boxes for two hours during the night period.

All the rats were weighed weekly, in order to adjust the oral medication dosage.

The animals in groups S.I and S.III received intra-articular injections of hyaluronic acid in the right knee once a week for five weeks starting at the end of the 12^th^ postoperative week (third month), and two more injections in the 25^th^ and 26^th^ postoperative weeks. The animals in group C.II received only the hyaluronic acid vehicle, but in accordance with the same schedule as those in groups S.I and S.III.

In order to receive the intra-articular injections, the animals were put into an anesthetic chamber that was supplied with 3% halothane and oxygen at a flow rate of 3 l/min. After becoming sedated, the animals were retrieved from the chamber and were prepared by means of shaving and antisepsis. The animals in groups S.I and S.III were injected with 0.04 ml of hyaluronic acid (Hyalgan/Polireumin; 2 ml = 600-800 kDa), and the group C.II animals were injected with 0.04 ml of the hyaluronic acid vehicle. The technique was performed with the knee flexed at 90 degrees. The needle was introduced perpendicularly to the skin and laterally to the patellar ligament, at the depression just above the joint line, in the direction of the intercondylar notch.

Oral diacerein (50 mg/kg in a dilution with 0.1 g/l of evaporated milk) was administered to the animals in groups S.II and S.III, daily from the third postoperative month to the seventh month, until one day before these rats were sacrificed (28^th^ week).

The animals were sacrificed by means of injection of intraperitoneal ketamine at a dose of 10 mg/kg in association with 0.8 mg/kg of diazepam.

After sacrifice, both knees were removed, placed in 10% formaldehyde solution and then sent for histological analysis. The knees were kept in this solution for one day and then demineralized in 7.5% nitric acid for three days. Longitudinal slides of the distal femur and proximal tibia were stained with hematoxylin and eosin. The pathologist was blinded to the groups under analysis. Semi-quantitative histological changes were recorded in accordance with a table of arthritic alterations that was designed by the pathologist and co-author (CRGCMO), based on the studies by Moskowitz et al.,^8^ Colombo et al.^2^^,^^14^ and Mazières et al.^16^ (Table 1). The arthritic grade was calculated by adding the points scored from the alterations seen on the slide.

**Table 1.**
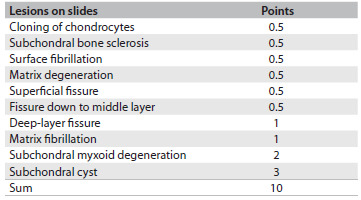
Scoring system used to sum degenerative changes

The final score defined the degree of arthritis in accordance with the following criteria: mild if the score was up to 2.5; moderate if the score was between 3 and 7; or severe if the score was between 7.5 and 10.

Animals that died before the end of the experiment, or that, from histological evaluation, presented any disease that would interfere with the osteocartilaginous metabolism, were excluded from the study.

Descriptive statistics on the ordinal (quantitative) sample values were calculated. The Mann-Whitney U-test was used in the comparisons between ordinal nonparametric and unrelated data, while the Wilcoxon test was used when the data were related (paired). The Kruskal-Wallis (variance analysis) test was used for comparison of more than two nonparametric strings. Pearson’s correlation and regression tests were performed between ordinal strings. The significance level of 5% (a = 0.05) was chosen.

## RESULTS

Seven animals died before the scheduled sacrifice date: three in group C.II and one each in groups C.I, C.III, C.IV and S.III. After sacrificing and slide analysis, one animal in group C.I was found to present plasmocytoma in both knees, and was excluded from the sample. A total of eight animals were excluded.

**Figure 2.**
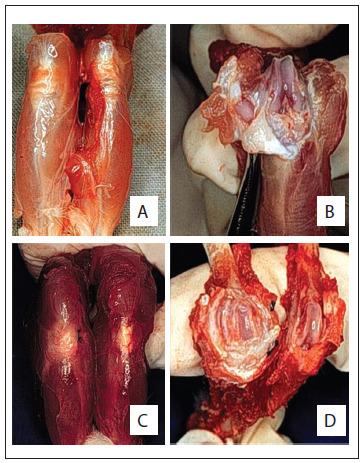
Macroscopic appearance of non-operated and operated knees of Wistar rats at 10 months of age. Non-operated (C.III group) external view (A) and internal view (B). Right operated knee and left non-operated knee (C.I group) external view (C) and internal view (D). Nylon stitches on the medial aspect of the knee.

**Figure 3.**
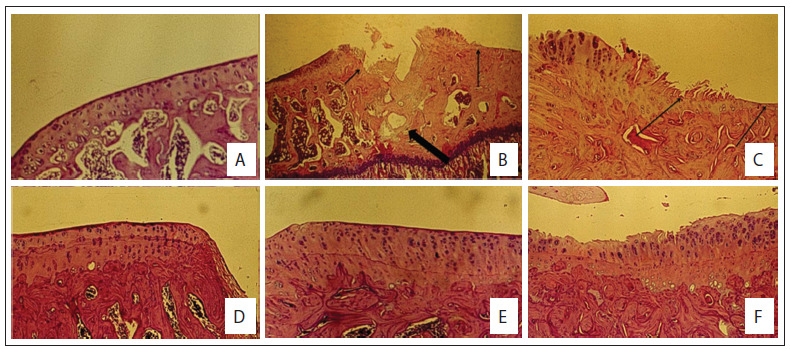
Histology of the control groups of Wistar rats. (A) Normal histology (100x, hematoxylin and eosin, HE); (B) Severe arthritis of the tibia with erosion (arrows) and subchondral cyst (large arrow) (C.I group; 25 x, HE); (C) Severe arthritis with deep fissure and erosion (arrows) (C.I group; 100 x, HE); (D) Mild arthritis of the femoral surface (C.II group; 25 x, HE); (E) Non-operated knee from control group C.III showing natural aging process and cloning of chondrocytes; (F) Tibial surface 12 weeks after meniscectomy (C.IV group; 100 x, HE).

**Figure 4.**
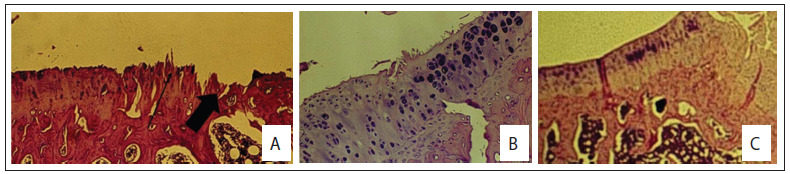
Histology of the study groups of Wistar rats. (A) tibial surface in S.I group with erosion (large arrow) and fibrillation (arrow); (B) Operated tibial condyle S.II group showing cloning of chondrocytes, matrix degeneration and superficial fissures; (C) Operated tibial condyle (S.III group) showing cloning of chondrocytes and matrix degeneration.

Figure 2 shows the external and intra-articular macroscopic evaluations on the non-operated and operated non-medicated knees, 28 weeks after inclusion. Figures 3 and 4 show slides from the control and study groups.

Tables 2, 3 and 4 summarize the degenerative changes found on the distal femur and proximal tibial of all groups. The proximal tibias of the meniscectomized knees had greater degenerative changes. Seven months (28 weeks) after surgery, the proximal tibia in the rats that were subjected to surgery but were not medicated showed a score corresponding to severe arthritis, i.e. 8.6 ± 2.3 (median 10; range 5 - 10, with a worst possible score of 10), while the group that received daily oral doses of diacerein from the third to the seventh postsurgical month scored 2 ± 2.9 (median 1; third quartile 2; range 0.5 - 10) in the proximal tibia. All the groups that were operated on showed a statistically significant difference in degenerative changes between the operated and nonoperated sides, on the proximal tibia.

**Table 2.**
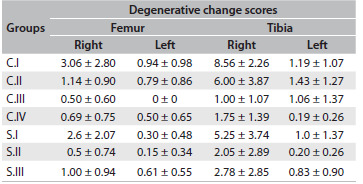
Means and standard deviations of degenerative change scores for the femur and tibia in all groups

C.I = operated, non-medicated; C.II = operated, injections of HA vehicle; C.III = non-operated, non-medicated; C.IV = operated, non-medicated, sacrificed three months post-meniscectomy; S.I = operated, receiving intra-articular HA injections; S.II = operated, oral diacerein from the third to the seventh postoperative month; S.III = operated, received both medications.

**Table 3.**
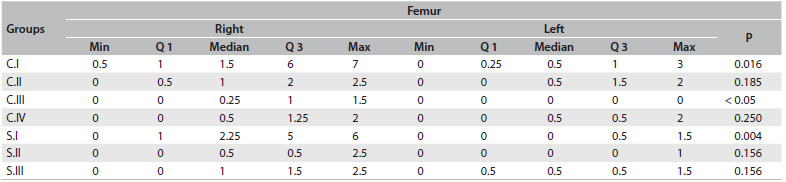
Median, minimum, maximum and first and third quartiles (Q1 and Q3) of femoral degenerative change scores in all groups: comparison between right and left sides using Wilcoxon test (α = 0.05)

C.I = operated, non-medicated; C.II = operated, injections of HA vehicle; C.III = non-operated, non-medicated; C.IV = operated, non-medicated, sacrificed three months post-meniscectomy; S.I = operated, receiving intra-articular HA injections; S.II = operated, oral diacerein from the third to the seventh postoperative month; S.III = operated, received both medications; Min = minimum; Max = maximum.

**Table 4.**
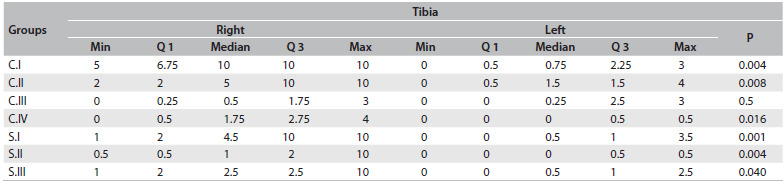
Median, minimum, maximum and first and third quartiles (Q1 and Q3) of tibial degenerative change scores in all groups: comparison between right and left sides using Wilcoxon test (α = 0.05)

C.I = operated, non-medicated; C.II = operated, injections of HA vehicle; C.III = non-operated, non-medicated; C.IV = operated, non-medicated, sacrificed three months post-meniscectomy; S.I = operated, receiving intra-articular HA injections; S.II = operated, oral diacerein from the third to the seventh postoperative month; S.III = operated, received both medications; Min = minimum; Max = maximum.

In Table 5, we compared the degenerative changes in all groups. The differences were significant on the right side (operated knees: right femur, P = 0.008; and right tibia P = 0.0001), between the groups. The degenerative changes (Tables 2, 4 and 5) to the tibias of rats that were subjected to operations but were not medicated and were sacrificed at 28 weeks scored 8.56 on average (median 10; severe arthritis). These results were significantly worse than those found in the tibias of rats sacrificed 12 weeks after surgery (score 1.75; mild arthritis by definition; P = 0.001) and significantly worse than those found in animals of the same age that were not subjected to operations (C.III; P = 0.001).

**Table 5.**
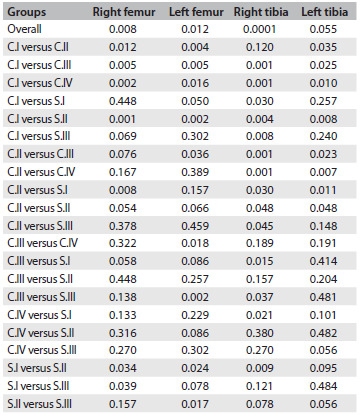
Comparison of degenerative change scores between groups for each side and bone end of the knee joint: overall comparison using Kruskal-Wallis test (α = 0.05) and comparison between group pairs using Mann-Whitney “U” test (α = 0.05)

C.I = operated, non-medicated; C.II = operated, injections of HA vehicle; C.III = non-operated, non-medicated; C.IV = operated, non-medicated, sacrificed three months post-meniscectomy; S.I = operated, receiving intra-articular HA injections; S.II = operated, oral diacerein from the third to the seventh postoperative month; S.III = operated, received both

Injection of hyaluronic acid diminished the degenerative changes found in the rat groups that were subjected to operations but not medicated (C.I x S.I; P = 0.03) and in the rat groups that were only injected with the vehicle of the hyaluronic acid (C.II x S.I; P = 0.03). Daily administration of oral diacerein from the 12^th^ to the 28^th^ week after surgery (S.II) showed significantly better results (average tibial degenerative changes of 2 points (median 1) on the right side and 0.2 (median 0) on the non-operated side) compared with those that were operated but not medicated (C.I) (P = 0.004 for the operated side and P = 0.008 for the non-operated side; Table 5). S.II showed no statistically significant difference in the results obtained from the two knees (distal femur and proximal tibia) of the animals that were sacrificed at 12 weeks after surgery (time point at which the medication was delivered to group S.II), P = 0.32 (right distal femur); P = 0.09 (left distal femur); P = 0.38 (right proximal tibia) and P = 0.48 (left proximal tibia); Table 5.

Furthermore, daily oral diacerein combined with intra-articular injection of hyaluronic acid did not show better results: P = 0.078 (proximal right tibia). Daily oral diacerein led to better results than injection of hyaluronic acid given in the third and sixth month after surgery (right tibia, P = 0.009; right femur, P = 0.34).

The initial animal weights ranged from 228.4 ± 27.1 grams (C.III) to 309.4 ± 51.6 grams (C.II) with no differences between the groups (P = 0.25). The final weights ranged from 470.1 ± 81.4 grams (C.IV) to 535.8 ± 55.8 (S.I) with no particular difference between the groups (P = 0.36).

None of the femoral changes correlated with the weight (either initial or final). The degenerative changes to the operated tibia, which were clinically more significant, showed no particular correlation with the animal’s weight at the beginning of the study. The only significant association found in the study was a correlation between final weight (FW) and degenerative changes (DG) in the right tibia in group S.I. The linear regression equation for this was DG = 32.28 - 0.05 x FW (P = 0.012).

## DISCUSSION

The main goal of this study was to provide an animal model of secondary OA that would be suitable for testing drugs that might retard or reverse cartilage degradation. For this purpose, we tested two specific drugs.

We proposed a new experimental model of secondary OA caused by total medial meniscectomy and ligament repair. In this model, young adult Wistar rats were subjected to total medial meniscectomy that had to be managed by means of sectioning and suturing the tibial collateral ligament. This is a slow but aggressive model of OA that leads to stiffness and severe degenerative changes, i.e. exposure of the subchondral bone and osteophyte formation seven months after the operation.

We chose rats mainly because of the ease of working with 70 animals of a smaller size, but also because studies on rabbits that remained confined to cages showed better results with disease-modifying drugs^17^ than studies on larger animals that were free to walk during the study.^5^^,^^18^ Therefore, in this study, we used small animals that were free to walk during the nighttime. Male rats were chosen in order to avoid the effects of female hormones on joint degeneration.^19^ According to Silberberg and Silberberg,^7^ the degree in which OA develops in a fat-rich diet model depends on the lineage, gender, age at the introduction of the diet and the length of time for which this diet is given. Thus, we tried to reduce the number of variables as much as possible, so as to have a homogeneous set of animals, which was represented by the similar weight results.

In Tables 2, 3 and 4, we summarized the results from the degenerative changes found in the right operated and left non-operated knees. It is well known that as few as four animals per group may suffice for screening if the test drugs are highly effective. Otherwise, eight or more animals may be required.^2^ Despite losses in almost all groups, we were able to establish the degenerative changes found three months after the operation (C.IV) and seven months after the operation (C.I), and also the natural aging process in the animals that did not undergo surgery (C.III).

In this study, animals of approximately 10 months of age (C.III) had mild degenerative changes in both knees due to cloning of chondrocytes, superficial fissures, surface fibrillation and, in some cases, subchondral bone sclerosis and matrix degeneration.

Animals that were subjected to surgery and sacrificed 12 weeks after meniscectomy, showed mild arthritis (mean and median score of 1.75) of the right knee (tibia) and almost no degenerative changes in the left knee (mean score = 0.19, median = 0), probably because the animals were young (between five and seven months of age) and because there had been little time for arthritis to affect the contralateral knee.^20^

The animals in the C.I group showed the worst degenerative changes (8.56 in the right tibia and 1.19 in the left tibia). The degree of arthritis in the operated knee was significantly worse than among the animals sacrificed at 12 weeks (C.IV) and among the animals that were not subjected to surgery (P = 0.001 for both, in the right tibia; Tables 4 and 5). The degree of arthritis on the left side in the C.I group was significantly different from that of animals that did not undergo surgery (C.III) (P = 0.001) but the difference was not clinically relevant. Both groups had mild arthritis: mean score 1.19, median 0.75, in C.I; and mean 1.06, median 0.25, in C.III. Unilateral inflammation can induce distal bilateral degeneration of articular cartilage through neurogenic mechanisms, thus suggesting involvement of neuropeptides.^20^ It is possible that if the study had been carried out for a longer period of time, a more relevant difference would have been shown between the non-operated side and the degenerative changes expected through aging.

One of the questions in the present study was whether injection of hyaluronic acid (500-730 kDa, 10 mg/ml) once a week for five weeks in the third postoperative month and then two more weekly injections^21^^,^^22^ in the sixth month (weeks 25 and 26) would delay or prevent degenerative changes in the operated knee. It is known that the half-life of hyaluronate in the joint is 13 hours, which leads to a residence time of about seven days in the joint.^21^ The two extra injections in the sixth postoperative month were based on the study by Schiavinato et al.,^22^ so as to limit the period without any medication to a maximum of 10 weeks.

Hyaluronic acid is known to have analgesic properties^23^ and has been shown in some animal studies to prevent degenerative changes and stiffness caused by immobilization, ligament instability and meniscectomy, depending on the timing, dose and molecular weight.^10^^,^^17^^,^^18^^,^^22^^,^^24^^,^^25^ In human studies,^26^^,^^27^ it has been shown to relieve pain and increase function, and it may potentially delay the structural progression of the disease.

In our study, the protection for the joint cartilage given through injection of hyaluronic acid (S.I group) was greater than what was achieved through injection of the vehicle for hyaluronic acid (Tables 2 to 5). Questions remain regarding the appropriate dose, frequency and type of hyaluronic acid that will yield better results. We injected 0.04 ml of hyaluronic acid or vehicle for hyaluronic acid. This dose is similar to the 0.1 ml/kg that were injected in rabbits or dogs in other studies.^17^^,^^18^^,^^28^^,^^29^ However, the results were extremely heterogeneous. This may be due to several reasons, such as the degree of arthritis when the dose is initially injected, whether intra-articular injection of the medication was accomplished and whether the animal responded to the medication. In reality, injection of any medication is feasible in a normal rat’s knee. Some animals, three months after surgery, already had stiffness and/or synovitis in the operated knee. Those animals were difficult to inject and had worse results. Three rats presented severe arthritis, scoring 10 points; three rats showed moderate arthritis scoring between 5 and 7; and four rats scored between 1 and 2 (mild arthritis). Table 4 shows the scores for the group S.1: first quartile (2), third quartile (10) and median (4.5). Table 3 shows the less pronounced femoral changes. Although on average injection of hyaluronic acid modified the course of post-meniscectomy osteoarthritis, this may not have been the best experimental model for testing intra-articular medications.

Because of the already-mentioned possibility that unilateral inflammation could induce distal bilateral degeneration of joint cartilage via neurogenic mechanisms,^20^ the idea of an oral medication with the possibility of disease-modifying effect is quite appealing. Diacerein (and its active derived metabolite rhein) is an anthraquinone disulfonic acid disease-modifying osteoarthritis drug. It has been shown *in vitro* to inhibit dose-dependent cathepsin B activity and time and dose-dependent interleukin-1-beta-stimulated proteoglycan release from the cartilage matrix; and *in vivo* to reduce all cartilage degeneration parameters and joint stiffness.^6^^,^^30^ It modulates the expression of matrix-degrading enzymes and cell proliferation of articular chondrocytes through inhibiting ERK and JNK-AP-1 dependent pathways.^31^ It downregulates pro-inflammatory cytokine expression (interleukin-1-beta (IL-1b), IL-12 and TNF-alpha).^32^

In this study, the animals that received 50 mg/kg of oral diacerein daily from the third to the seventh postoperative month showed the best results on all slides (femur and tibia bilaterally, whether operated on or not). There was no difference between the findings seven months after surgery in the group that received daily doses of diacerein and the group that was sacrificed three months after the operation. Diacerein prevented progression of osteoarthritis in the operated knee and the normal age-related or even neurogenic mediated arthritis of the contralateral side (Tables 2 to 5).

The combination of oral diacerein and injection of hyaluronic acid did not improve the results obtained through an oral dose of diacerein alone (Tables 2 to 5).

These results were not affected by the animal’s weight. However, none of the animals were obese and they all had similar weights.

The meniscectomy osteoarthritic model in rats proved to be a useful model for testing osteoarthritis disease-modifying drugs. The limitations of the study relate mainly to the histological analysis, which was mostly done using hematoxylin-eosin, thereby missing early matrix changes, and the short follow-up. Future goals would include improvement of histological analysis and testing of other disease-modifying osteoarthritic drugs.

## CONCLUSIONS

1) The meniscectomy experimental model in rats presented here was effective in producing mild arthritis three months postoperatively and severe arthritis seven months postoperatively; 2) oral diacerein was effective in reducing the degenerative changes in both knees; 3) there was some protection for joint cartilage through intermittent intra-articular injection of hyaluronic acid; 4) using the two drugs in association did not improve the results.
